# Development of the Enamel

**Published:** 1882-02

**Authors:** M. S. Dean

**Affiliations:** Chicago


### SELECTIONS.

### DEVELOPMENT OF THE ENAMEL.

BY DR. M. S. DEAN, D. D. S.,
CHICAGO.

While the epithelial nature of the enamel has become a settled question, the *mode* of its production is still a matter of controversy. Indeed, physiologists are pretty evenly divided as to whether the enamel-cells are actually *converted* into enamel-prisms, or whether the enamel is the *product* of these cells.

On the one hand we have Schwann, Tomes, Waldeyer, Frey, and others, who hold that the enamel is formed by an actual conversion of the enamel-cells (Tomes)---or, in other words---that the enamel may be regarded as the *petrified* dental epithelium (Waldeyer.)

On the other hand we have Huxley, Lent, Kolliker, Magitot, Kollmann, and many others, who believe the enamel to be the product of a secretion or an *exudation* of the enamel-cells.

Although the advocates of the latter theory do not perfectly agree as to the manner in which this *exudation* or *secretion* takes place, yet in this there seems to be no very *essential* difference.

Huxley claims the existence of a preformative membrane, which clothes the free surface of the internal epithelium. This, according to his theory, lies between the enamel-organ and the dentine-germ *until* the formation of the enamel commences; but afterwards *between* the enamel-cells and the forming enamel, so that in any stage of development this membrane may be raised from the external surface of the new enamel; and finally, after the enamel is completed, this membrane becomes the *cuticula dentis*, or Nasmyth's membrane.

Histologically, it seems to me that Dr. Magitot's description may be made to harmonize perfectly with the above; though he believes the *continuity* of this membrane is effected by artificial means.

He asserts that the basal extremity of the enamel-cells, *i. e.*, the ends that are presented to the dentine-germ, are closed by individual membranous *opercula*; that the opercula may be stripped off from the ends of each one of these cells. But, that in some preparations which have been affected by reagents, a ribbon can be detached from a row of these cells which is composed of several of these opercula united together so as to *apparently* form a continuous membrane. This would, of course, conform to Huxley's *membrana præformativa*. Now, as to the formation of the enamel, Dr. Magitot says: The enamel-prisms are formed directly from a sort of *exudation* produced by each cell, which passes through the little opercule that closes or covers its extremity. The calcification of this exudation takes place upon the dentine-cap, and between it and the opercule. Each enamel-cell thus forms a complete prism.

From this it may be seen that these membranous opercula constantly intervene between the forming enamel and the enamel-cells, and might be raised from the surface of the former as a continuous membrane by the action of reagents. So I see no conflict in the histological descriptions of Huxley and Magitot, except that in one this membrane is naturally *continuous*, and in the other it is *made so* by the artificial union of the many opercula.

If I understand the theory of Kolliker and his school correctly, it does not materially differ from the above. He considers the enamel the product of the secretion of the enamel-cells, which exudes from the basal extremities, each cell forming an enamel-prism. Kollman is also in accord with these authors as to the enamel being a hardened excretion of the cells, and claims that the free extremities of these cells are invested with a membrane which afterwards becomes the *cuticula dentis*.

This is a brief and, I believe, a tolerably correct synopsis of the views entertained by a few of the leading histologists whose opinions upon this subject have been most prominent.

Tomes, father and son, ably defined their theory that the enamel-cells become calcified, and, though they admit that a membrane may be raised from the surface of the young enamel, they consider it purely an artificial product, and the result of the action of acids on the most recently calcified portions of the enamel-cells. The histological facts that they adduce in support of the artificial character of this membrane, if they are admitted, it seems to me, are conclusive, and fatal to the theory that a true membrane exists between the forming enamel and the enamel-cells. For if the enamel-cells that are removed from the developing enamel are correctly figured by these authors, *i. e.*, if long processes are drawn out from the axial portion of the calcifying prism; and if the membrane which may be raised from the developing enamel is regularly perforated with holes corresponding to the enamel-prisms; and if, again a horizontal section taken from the forming enamel after the cells have been removed, present a foraminated appearance; if all these are established histological facts, and I am not aware that they are disputed, then it seems to me that of the two theories, the actual conversion of the cells by calcification inte enamel, is far the best supported by facts. It would appear to me that the whole question hinges upon the fact of whether this is a *true* or an *artificial* membrane, and until this is determined by chemico-histology, the physiological phenomena that take place in the formation of the enamel must be conjectural.

But whether the enamel-cells are actually converted into enamel prisms, or whether the latter are the calcified secretion of these cells, the question naturally arises, and the answer must be the same in *either case*, "Through what media are the lime salts obtained, of which the enamel is mainly composed?" This is an important physiological point, and one that is immediately connected with the question just discussed.

With the exception of Beale and Castanie, who claim to have found capillaries in the *Stratum Intermedium*, histologists, so far as I know, are either silent in regard to the presence of vessels in the enamel-organ, or they deny their existence altogether. Though this is *primarily* an anatomical question, it has also its physiological bearings on the development of the enamel. For, notwithstanding the general belief that the gelatinous mass, which forms the greater portion of the enamel-organ, filling up the interstices between the stellate cells, "has no more important function to perform than to fill up the space which is to be subsequently occupied by the growing tooth," it would seem to me *more than probable* that this proteine substance is a store-house of phosphate of lime, which stands ready to yield its supplies whenever called upon by the enamel-cells. This I believe would be in accordance with known chemical and physiological laws. This gelatinous or albuminous mass would, of course, receive fresh supplies of calcareous material from the rich plexus of vessels which surround the enamel-organ. But it would always hold a supply in *reserve*, which might be drawn upon in case the external sources should be temporarily interrupted by exanthematous affections, or other causes.

Gentlemen, knowing your partiality for short papers, I have tried to comply with your wishes. If you are not tired of this subject, I will, during the discussion, attempt to explain the subject more fully, by the aid of the pictures which hang upon the wall.

DISCUSSION.

DR. TAYLOR: Is the enamel nourished in any degree from the pulp?

Dr. Dean: Taking the question as it has been put, I would unhesitatingly answer it in the affirmative; *i. e.*, that the *formed* enamel is undoubtedly nourished, or at least its disintegration is in some degree held in check by the plasma from the dentinal pulp. But whether this is effected by a physiological or by a physical process is a question that I am not so ready to attempt to answer. That the dentinal pulp does not contribute anything to the *development* of the enamel, is most certain. The enamel, as has been shown, is entirely an epithelial product.

Dr. Black: I do not know just what to say about this. It is intimately connected with other questions relating to development, and I am not certain whether I can make myself understood. I will try to answer Dr. Taylor's question. The paper directed attention to the basement membrane, which always underlies the epithelial tissues. The enamel and the dentine are therefore always separated by the basement membrane. The blood vessels, even the smallest capillaries, never pass this membrane. The epithelial tissues must therefore be wholly nourished by endosmotic circulation. The dentine is calcified from without inward, the external portion being hardened first, but the enamel is calcified from within outward, the *internal* portion first, so that if we suppose the forming enamel to receive its nourishment from the pulp, it has the hard layer of formed enamel (as well as dentine) between its supply of nourishment and the growing cells, and the transfer of nourishment in sufficient quantity would be impossible. The special provision for the nourishment of the forming enamel is in the interior of the enamel organ, which is filled with stellate cells, whose very large inter-spaces are filled with the fluid which furnishes the food to the ameloblasts out of which to build the enamel. This is the only place in which epitheliel cells ever become stellate.

In the last five or six years I have spent a great deal of time in studying cancer, which is an erratic growth of epithelial tissue, yet it never penetrates beneath the basement membrane, and if the cancer cells stray away into the blood vessels, or lymphatics, and begin to develop a cancerous growth in a new place, the basement membrane is also developed about the growing group of cells, as it were dividing them from the connective tissue amidst which they grow. We have seen this exemplified in so many cases and so many diverse localities that we are satisfied of its constancy. These cells are thus hedged away from the connective tissue cells, and wandering into the surrounding tissues, or the blood or lymph streams, only occurs in case of the solution of this membrane by different portions of the erratic growth impinging upon each other, or breaks caused by motion operating upon the indurated tissue.

The common earth worm furnishes a ready means for studying the epithelial tissues with its basement membrane, under the microscope. The dermal epithelium cells are very large ; several times larger than the columnar layer in man.

Dr. Taylor : Dr. Garrettson says that the enamel gets its supply of nourishment from the pulp vessels, and from the vessels of the periostium and cementum, and the apical vessels.

I would like to ask Dr. Dean to tell what Nasmyth's membrane is ?

Dr. Dean : This is a question that is closely allied to the development of the enamel, according to several authorities, and hence its discussion will not be inappropriate here. Nasmyth's membrane, or the *cuticula dentis*, is, I believe, an epithelial product—not a calcified one, as the enamel is—but cornified epithelium, like the nails. This is, I think, shown both by its chemistry and its histology. There is, however, a difference of opinion in regard to the origin of this tissue, and it is only fair that I give you your choice of the principal theories concerning it.

Waldeyer, Kolliker, and other German authors, are the main supporters of the theory that the *cuticula dentis* originates from the cells of the enamel organ; the first of these authors believing that, after the enamel is formed, the cells that constitute the external epithelium of the enamel organ, with a few additional cells of the *stratum intermedium*, become cornified and constitute the Nasmyth membrane. He failed to find lime salts in its composition, and in burning, its odor was that of horn. Kolliker's theory differs from Waldeyer's in this: He says that when the enamel is completely formed, the internal epithelium (the ameloblasts), furnish another continuous layer, that envelops the whole.

Tomes, Magitot, Wedl, and others, consider it to be the continuation of the cement, a rudimentary osseous tissue; in which case it must be derived from the connective-tissue elements. The facts brought forward by Prof. Tomes strongly favor this theory. He finds lacunal cells in the thicker portions of this membrane, and believes it to be continuous with the cement.

Huxley, as has already been stated, believes the *cuticula dentis* to be a transformation of the *membrana prœformativa.* To accept this theory, one must adopt his views regarding the development of the enamel.

These are the principal theories regarding the origin of this membrane, the parentage of which is disputed by the *epiblastic* and *mesoblastic* germ layers. I cannot give my reasons fully here for believing that it belongs to the former, but may attempt this some other time. Specimens of this cuticle may be most easily procured from the teeth of very young lambs.

Dr. Black asked Dr. Brophy if the last edition of Gray's Anatomy was not incorrect and antiquated in what it says about the development of the teeth.

Dr. Brophy: I have said, and I believe, that Gray's Anatomy is the third book in the world (the Bible and Shakespeare taking precedence): but it does contain a number of errors, notably that one in regard to the anastomosis of the coronary arteries of the heart, and *all* that portion relating to the development of the teeth, is entirely untrue and misleading.—*Trans. Ill. State Dental Society.*

